# Oligodendrocyte-Specific STAT5B Overexpression Ameliorates Myelin Impairment in Experimental Models of Parkinson’s Disease

**DOI:** 10.3390/cells14151145

**Published:** 2025-07-25

**Authors:** Yibo Li, Zhaowen Su, Jitong Zhai, Qing Liu, Hongfang Wang, Jiaxin Hao, Xiaofeng Tian, Jiamin Gao, Dandan Geng, Lei Wang

**Affiliations:** 1 Department of Human Anatomy, Hebei Medical University, Shijiazhuang 050017, China; liboya0108@163.com (Y.L.); suzhaowendoc@outlook.com (Z.S.); zjthahahahah@outlook.com (J.Z.); whf19970212@163.com (H.W.); h13782717807@126.com (J.H.); 15230182715@163.com (X.T.); gjm13643429107@163.com (J.G.); 2 The Key Laboratory of Neural and Vascular Biology, Ministry of Education, Hebei Medical University, Shijiazhuang 050017, China; liuqingdiyouxiang@163.com; 3 Neuroscience Research Center, Hebei Medical University, Shijiazhuang 050017, China; 4 Hebei Key Laboratory of Neurodegenerative Disease Mechanism, Shijiazhuang 050017, China

**Keywords:** Parkinson’s disease, oligodendrocyte, substantia nigra, single-nuclear RNA sequencing, myelin sheath, methylation

## Abstract

**Background:** Parkinson’s disease (PD) involves progressive dopaminergic neuron degeneration and motor deficits. Oligodendrocyte dysfunction contributes to PD pathogenesis through impaired myelination. **Methods:** Single-nucleus RNA sequencing (snRNA-seq) of PD mice revealed compromised oligodendrocyte differentiation and *STAT5B* downregulation. Pseudotemporal trajectory analysis via Monocle2 demonstrated impaired oligodendrocyte maturation in PD oligodendrocytes, correlating with reduced myelin-related gene expression (*Sox10*, *Plp1*, *Mbp*, *Mog*, *Mag*, *Mobp*). DoRothEA-predicted regulon activity identified STAT5B as a key transcriptional regulator. **Results:** Oligodendrocyte-specific *STAT5B* activation improved myelin integrity, as validated by Luxol Fast Blue staining and transmission electron microscopy; attenuated dopaminergic neuron loss; and improved motor function. Mechanistically, *STAT5B* binds the *MBP* promoter to drive transcription, a finding confirmed by the luciferase assay, while the DNMT3A-mediated hypermethylation of the *STAT5B* promoter epigenetically silences its expression, as verified by MethylTarget sequencing and methylation-specific PCR. **Conclusions:** DNMT3A inhibited the expression of *STAT5B* by affecting its methylation, which reduced the transcription of *MBP*, caused oligodendrocyte myelin damage, and eventually led to dopamine neuron damage and motor dysfunction in an MPTP-induced mouse model. This DNMT3A-*STAT5B*-*MBP* axis underlies PD-associated myelin damage, connecting epigenetic dysregulation with oligodendrocyte dysfunction and subsequent PD pathogenesis.

## 1. Introduction

Parkinson’s disease (PD), the second most common neurodegenerative disorder, is classically defined by the progressive degeneration of nigrostriatal dopaminergic neurons and subsequent motor impairments [1,2]. While neuronal pathology has dominated PD research, emerging evidence implicates oligodendrocyte dysfunction as a critical contributor to disease progression [3,4]. These glial cells not only maintain axonal integrity via myelination but also support neuronal survival through metabolic coupling. Clinical observations reveal that PD patients often exhibit white matter lesions (WMLs) in cortical regions, which correlate with the severity of motor symptoms [5]. Furthermore, recent whole-brain connectivity omics analyses have demonstrated reduced myelin content in the brains of PD patients, particularly in circuits associated with dopaminergic neurons [6], indicating that myelin disruption may impair dopaminergic signaling [7,8]. However, the molecular mechanisms driving oligodendrocyte impairment in PD remain elusive.

Signal transducer and activator of transcription 5B (STAT5B), a pleiotropic transcription factor regulating cell survival and differentiation [9,10], has demonstrated neuroprotective effects in neurodegeneration [11,12,13]. In spinal muscular atrophy, STAT5 activation preserves motor neuron function [14]. STAT5B deficiency reduces striatal dopamine levels and exacerbates dopaminergic neuron loss [15]. Critically, our reanalysis of single-nucleus RNA sequencing (snRNA-seq) data from an MPTP-induced mouse model revealed the selective downregulation of *STAT5B* transcriptional activity in substantia nigra (SN) oligodendrocytes, with STAT5B expression positively correlated with myelin integrity. However, the mechanism by which oligodendrocytic STAT5B regulates myelin maintenance and dopaminergic neuron survival in PD remains unclear.

In this study, we demonstrate that restoring STAT5B expression in oligodendrocytes rescues myelination defects and motor dysfunction in PD models. Mechanistically, STAT5B contributes to myelin damage by transcriptionally inhibiting myelin basic protein (MBP) expression. Furthermore, we elucidate DNA methyltransferase 3A (DNMT3A)-mediated hypermethylation at the *STAT5B* promoter (position 2202) as the key epigenetic mechanism underlying decreased *STAT5B* transcription in PD.

By uncovering the DNMT3A-*STAT5B*-*MBP* axis in PD pathogenesis, we reveal how oligodendrocyte epigenetic dysregulation drives dopaminergic circuit failure, propose *STAT5B* reactivation as a dual-target therapy for myelin repair and neuroprotection, and nominate *STAT5B* promoter methylation as a translatable progression biomarker.

## 2. Materials and Methods

### **2.1. Establishment of MPTP-Induced Mouse Model** 

To establish an animal model of PD, 8-week-old male C57BL/6 mice were intraperitoneally (i.p.) administered 1-methyl-4-phenyl-1,2,3,6-tetrahydropyridine (MPTP) (Macklin, catalog number: M832929, Shanghai, China) at a daily dose of 30 mg/kg body weight for five consecutive days (PD group). The control group received an equivalent volume of physiological saline. Behavioral assessments were performed to evaluate and compare the physical activity levels of both groups. All animal procedures were conducted in accordance with the Animal Welfare Act, as well as the National Institutes of Health Guide for the Care and Use of Laboratory Animals (NIH Publication No. 85–23, revised 1996), and received approval from the ethics committee at Hebei Medical University (IACUC-Hebmu-P-2023157). Animals with pre-existing health conditions were excluded from the study. All researchers involved in the study were blinded to group assignments.

### **2.2. Mouse snRNA-Seq Data Processing** 

The data were processed using Seurat (v4.2.2) following standard pipelines. After quality control (excluding cells with >20% mitochondrial genes), we performed uniform manifold approximation and projection (UMAP)-based clustering (resolution = 0.5) to identify 25 initial clusters. Cell types were annotated using marker genes (identified by FindAllMarkers function) and cross-referenced with the CellMarker and PanglaoDB databases. Oligodendrocytes were subsequently subclustered for detailed analysis. For differentially expressed gene (DEG) analysis, we compared the MPTP-induced PD and control groups using FindMarkers (thresholds: *p* < 0.05, |log2FC| > 0.5). Significant DEGs in oligodendrocytes underwent functional enrichment analysis via clusterProfiler (v3.18.4), with false discovery rate (FDR) correction for multiple testing.

### **2.3. Analysis of the Whole Mouse SN Dataset** 

#### 2.3.1. Inference of Pseudotime Ordering Using Monocle2

To elucidate alterations in oligodendrocyte cellular states between the PD and control groups, we employed Monocle2, a robust algorithm for the reconstruction of cellular state trajectories after subclustering oligodendrocytes. This analysis critically involved identifying genes that potentially influenced the observed cellular state changes between the PD and control groups and their associations with the cellular state trajectory.

#### 2.3.2. DoRothEA-Predicted Regulon Activity

To explore changes in transcription factor (TF) activity in oligodendrocytes between the PD and control groups, we utilized the DoRothEA (v3.21) R package [16]. DoRothEA is a gene regulatory network (GRN) resource that deduces TF activity from gene expression data by analyzing TF–target gene interactions. We applied DoRothEA with confidence levels A, B, and C to compute viper scores, which quantified differential TF activity between the PD and control groups.

### **2.4. Mouse Brain Stereotactic Injection of the SN** 

Mice were anesthetized with isoflurane and immobilized in a stereotactic frame. After scalp disinfection and incision, we targeted the SN using coordinates relative to bregma and lambda (AP: −3.11 mm, ML: ±1.4 mm, DV: −4.2 mm). Adeno-associated virus (AAV) was delivered through a drilled craniotomy, with the needle maintained for 5 min post-injection before gradual withdrawal. The scalp was sutured and mice received standard postoperative care. Viral expression and behavioral outcomes were assessed 21 days post-injection.

### **2.5. Immunofluorescence Staining** 

Immunofluorescence was performed on cultured cells and 10 μm frozen substantia nigra (SN) sections. After PBS washes, sections were blocked with 5% donkey serum in PBS-T (0.5% Triton X-100) for 60 min at room temperature. Primary antibody incubations (4 °C overnight) included the following: Olig2 mouse monoclonal (1:300, sc-515947, Santa Cruz Biotechnology, Shanghai, China), STAT5B rabbit (1:200, CSB-PA022815LA01HU, CUSABIO, Wuhan, China) and mouse (1:200, 66427-1-Ig, Proteintech, Wuhan, China) monoclonals, and MBP rabbit monoclonal (1:200, 10458-1-AP, Proteintech, Wuhan, China). Sections were then incubated with Alexa Fluor-conjugated secondary antibodies (1:500, Thermo Scientific™, Shanghai, China) for 2 h: donkey anti-rabbit IgG-Alexa Fluor 594 (A-21207), donkey anti-mouse IgG-Alexa Fluor 488 (A-21202). After DAPI mounting, images were acquired using an Olympus FV1200 microscope (Olympusm, Tokyo, Japan) and analyzed with ImageJ (v1.51).

### **2.6. Immunohistochemical Staining** 

Mouse SN paraffin sections (5 μm thick) underwent dehydration through an ethanol series, followed by antigen retrieval using citrate buffer to facilitate immunohistochemical staining. Section processing strictly followed the manufacturer’s guidelines, as outlined in the UltraSensitiveSP Kit (catalog number: KIT-9720, MXB Biotechnologies, Fuzhou, China). To inhibit endogenous peroxidase activity, sections were treated with 0.3% hydrogen peroxide and subsequently incubated with fetal bovine serum (FBS) at 37 °C for 30 min. Following this, sections were incubated overnight at 4 °C with an anti-TH rabbit monoclonal antibody (1:200, catalog number: PB9449, Boster, Wuhan, China) and an anti-NfL mouse monoclonal antibody (1:300, catalog number: sc-71678, Santa Cruz Biotechnology, Shanghai, China). Image analysis was performed using the Image-Pro Plus 6.0 software.

### **2.7. Luxol Fast Blue Staining** 

Paraffin sections (5 μm thick) were deparaffinized in xylene. Sections were then stained overnight at room temperature with Luxol Fast Blue (LFB, catalog number: G3242, Solarbio, Beijing, China). After staining, they were washed with 95% ethanol and distilled water. Differentiation was performed using a Fast Blue differentiation solution for 15 s, followed by a 30 s treatment with 70% ethanol until a clear distinction between gray and white matter was achieved. The sections were subsequently washed with distilled water, quickly dehydrated in 95% and 100% ethanol, cleared in xylene, and mounted. Slides were air-dried for one week prior to imaging. Images were captured using an OLYMPUS BX53 microscope (Olympusm, Tokyo, Japan). The average optical density (AOD) was measured using the ImageJ (v1.51) software.

### **2.8. Transmission Electron Microscopy** 

Mice were perfused with freshly prepared electron microscopy perfusion solution, ensuring that tissue collection was completed within one minute post-euthanasia. The extracted brain tissue was promptly placed in a low-temperature ice box. Electron microscopy fixative was uniformly applied to the tissue surface, and a 1 × 1 × 1 mm^3^ tissue block, essential for effective fixative penetration, was carefully excised with a blade. The excised samples were then stored overnight at 4 °C. The following day, the samples were dispatched to the Transmission Electron Microscopy Laboratory at Hebei Medical University’s Large Instrument Experiment Platform for further experimental procedures. Myelin sheath observations in the SN of the mice were conducted using a HT7800 transmission electron microscope (Hitachi, Tokyo, Japan).

### **2.9. Cell Culture and Differentiation** 

The MO3.13 human oligodendroglia cell line (catalog number: CL-0772, Wuhan Pricella Biotechnology Co., Ltd., Wuhan, China) was cultured in high-glucose Dulbecco’s Modified Eagle’s Medium (DMEM) (catalog number: 11965092, Gibco, Shanghai, China) supplemented with 20% fetal bovine serum (FBS, catalog number: FB15015, Clerk, VA, USA) and 1% penicillin/streptomycin solution (catalog number: P1400, Solarbio, Beijing, China) in an H_2_O-saturated 5% CO_2_ atmosphere at 37 °C. For differentiation, once cells reached 70–80% confluency in poly-L-lysine-coated 24-well or 6-well plates, they were shifted to a serum-free differentiation medium containing 100 nM phorbol myristate acetate (PMA, catalog number: HY-18739, MedChemExpress, Shanghai, China). The medium was changed every 3 days, and cells were cultured for 7 days to induce differentiation [17]. Following the induced differentiation period, cells were subjected to drug intervention, transfection, and subsequent protein and RNA collection.

The SH-SY5Y human neuroblastoma cell line was cultured in DMEM/F12 medium (catalog number: 11320033, Gibco, Shanghai, China) supplemented with 10% FBS and 1% penicillin/streptomycin solution. The cells were maintained in an H_2_O-saturated 5% CO_2_ atmosphere at 37 °C. SH-SY5Y cells were differentiated by treatment with 10 μM retinoic acid (RA; catalog number: HY-14649, MedChemExpress, Shanghai, China) in serum-free or low-serum medium for 3–5 days. Following differentiation, neuronally differentiated cells were co-cultured with differentiated MO3.13 cells and subjected to LFB staining. Both cell lines were recently authenticated by STR profiling and tested for mycoplasma contamination, with all results being negative.

### **2.10. CCK-8 Assay** 

Cell viability was evaluated using the CCK-8 assay (catalog number: K1018; APExBIO, Shanghai, China). Briefly, 5 × 10^3^ cells/well were plated into 96-well plates and incubated for 24 h. Subsequently, cells were exposed to 1-methyl-4-phenylpyridinium iodide (MPP^+^) for various time intervals: 4, 6, 12, 24, and 48 h. After the treatment, 10 µL of CCK-8 reagent was added to each well, and cells were further incubated for 1 h. Absorbance (optical density) was recorded at 450 nm using a microplate reader. This experiment was performed in triplicate, with three wells per experimental condition. Data were analyzed using SPSS, and comparisons between treatment groups were conducted to identify significant differences, with a *p*-value < 0.05 considered statistically significant.

### **2.11. Transfection of Plasmids** 

The plasmids used in this study were constructed by GenScript. For plasmid extraction, *Escherichia coli* was cultured in LB liquid medium, and plasmids were extracted using a plasmid extraction kit from TIANGEN (catalog number: DP123, Beijing, China). The extraction process involved cell collection, resuspension, lysis, precipitation to remove impurities, washing, centrifugation, and elution, followed by plasmid concentration determination. For transfection, when the cells reached 60–70% confluency, plasmids and UltraFection 3.0 transfection reagent (catalog number: ELS300, SiZhengBai, Suzhou, China) were prepared and mixed and then incubated. The medium was not changed within 24 h after plasmid addition. qRT-PCR was performed 24 h post-transfection, and Western blot analysis was conducted 48 h post-transfection.

### **2.12. Transfection of siRNA** 

The siRNA constructs used in this study were provided by GenePharma. MO3.13 cells were passaged in 6-well plates and induced to differentiate before siRNA transfection. The CALNP™ RNAi in vitro transfection kit (catalog number: DN001-10, D-Nano Therapeutics Technology Co., Ltd., Beijing, China) was used for this purpose. A total of 5 µL of 20 µM siRNA solution was mixed with 40 µL of Solution A and 10 µL of Solution B, followed by the addition of 145 µL of cell culture medium, resulting in a total volume of 200 µL, which was then added to the 6-well plate containing 2 mL of the culture medium. The medium was not changed within 24 h after siRNA addition. qRT-PCR was performed 24 h post-transfection, and Western blot analysis was conducted 48 h post-transfection.

### **2.13. RT-qPCR** 

Total RNA was extracted using the Total RNApure Reagent Kit (ZP401, ZOMANBIO, Beijing, China), and 1 μg RNA was reverse-transcribed using HiScript III RT SuperMix (R323, Vazyme, Nanjing, China) under the following conditions: 42 °C (2 min), 37 °C (15 min), and 85 °C (5 s). RT-qPCR was performed with ChamQ Universal SYBR qPCR Master Mix (KT201, Vazyme, Nanjing, China) on a QuantStudio™ 6 Flex system (4484642, Applied Biosystems, Shanghai, China) under standardized cycling conditions: 95 °C (15 min), followed by 40 cycles of 95 °C (10 s), 60 °C (30 s), and 72 °C (20 s). GAPDH served as the endogenous control, and the relative mRNA expression was calculated using the 2^−ΔΔ^Ct method. All reactions were performed in triplicate, with statistical analysis conducted using two-tailed *t*-tests and one-way ANOVA. Primer sequences are provided in Table S1.

### **2.14. Western Blot** 

For protein sample preparation, the cells or mouse SN tissue were homogenized or lysed using RIPA buffer (catalog number: R0020, Solarbio, Beijing, China), which was augmented with PMSF 100 mM (catalog number: P0100, Solarbio, Beijing, China). Following ultrasonication, the samples were incubated on ice for 1 h. Protein solutions were clarified by centrifugation at 12,000× *g* for 20 min at 4 °C, and the supernatant was collected. The protein concentration was then determined using a BCA protein assay reagent kit (catalog number: PC0020, Solarbio, Beijing, China) and measured with an Infinite F200 plate reader (TECAN, Switzerland). The protein samples were combined with a 5× loading buffer (catalog number: P1040, Solarbio, Beijing, China). After heating, 20 μg of protein was resolved on a 10% SDS polyacrylamide gel, which was then electrotransferred onto polyvinylidene fluoride membranes. The membranes underwent blocking in 5% milk dissolved in TBST (0.1% Tween-20) for one hour before incubation with the following primary antibodies: rabbit anti-STAT5B (1:2000, catalog number: CSB-PA022815LA01HU, CUSABIO, Wuhan, China), mouse anti-GAPDH (1:5000, catalog number: GB120025, Servicebio, Wuhan, China), rabbit anti-MBP (1:200, catalog number: 10458-1-AP, Proteintech, Wuhan, China), and rabbit anti-TH (1:2000, catalog number: A5079, ABclonal, Wuhan, China). Subsequently, membranes were incubated with HRP-conjugated AffiniPure goat anti-rabbit IgG (H+L) (1:5000, catalog number: BF03008, Biodragon, Suzhou, China) and HRP-conjugated AffiniPure goat anti-mouse IgG (H+L) (1:5000, catalog number: SA00001-1, Proteintech, Wuhan, China), along with an Enhanced/Super ECL Kit (1:1, catalog number: BF06053-100, Biodragon, Suzhou, China). For statistical analysis, either two-tailed *t*-tests (for two groups) or one-way ANOVA (for three or more groups) were applied.

### **2.15. Animal Behavioral Experiments** 

#### 2.15.1. Rotarod Experiment

The rotarod assay was performed utilizing a mouse rotarod fatigue testing apparatus. The methodology was as follows. Each mouse was initially positioned onto the rotarod, which was set to an adjustable rotation speed of 25 revolutions per minute (rpm). A timer automatically documented the elapsed time from placement until the mouse dislodged from the rod (latency to fall). To ensure result reliability, a single measurement was conducted daily. By evaluating the duration of the mouse’s traversal on the rotarod, variations in motor performance were effectively analyzed.

#### 2.15.2. Climbing Pole Experiment

Mice were placed head-up on a textured wooden sphere attached to a cylindrical pole within their home cage. Motor performance was quantified by measuring the turning around time and pole climbing time (T). The time taken for the mouse to navigate from the wooden sphere to the wooden pole while maintaining a downward head orientation was denoted as T1, measured with a stopwatch. Upon the mouse’s arrival at the lowest point of the pole, this time was noted as T2. The time taken to climb from the sphere to the pole base (T = T1 [initiation] − T2 [completion]) was recorded via stopwatch. Each mouse completed two trials, with the mean climbing duration used for analysis. The rough surface ensured a consistent grip during testing.

#### 2.15.3. Gait Analysis

Gait assessment was conducted using an automated gait analysis system (Zhongshidi Technology Development Co., Ltd., Beijing, China). The mice were positioned at one terminus of the runway and permitted to traverse to the opposing end, where they could enter a dark enclosure. Each mouse underwent two housing sessions prior to the commencement of the formal experiment. The parameters measured encompassed the stride length, step velocity, and limb support time.

### **2.16. Bioinformatic Screening of Downstream Genes Using JASPAR and hTFtarget Databases** 

Potential downstream targets of STAT5B were identified through the systematic analysis of transcription factor binding sites (TFBSs) using the JASPAR and hTFtarget databases. Genes with significant associations (Q-value < 0.05) were retained after initial screening with an 80% relative score threshold. Four key parameters were normalized (0–1 range) and integrated into a composite score: the human TFBS count and maximum score (weight: 0.3 and 0.4, respectively); the mouse TFBS count and maximum score (combined weight: 0.3). The final prioritization considered both human data dominance and cross-species conservation evidence. This hierarchical approach not only prioritized human data but also integrated cross-species conservation evidence, thereby allowing for more comprehensive gene prioritization.

### **2.17. Luciferase Reporter Gene Assay** 

Here, 293T cells at ~60% confluence in 96-well plates were transfected in quintuplicate. After 24 h, luciferase activity was measured using the Dual-Luciferase^®^ Reporter System (E1910, Promega, Beijing, China). Cells were PBS-washed, lysed with 50 μL passive lysis buffer/well (12 min dark agitation), and transferred to opaque plates. Firefly luciferase signals were quantified and then quenched with Stop & Glo^®^ Reagent (50 μL/well) prior to Renilla luciferase measurement for normalization.

### **2.18. Actinomycin D Assay** 

MO3.13 cells were maintained in 6-well plates prior to treatment. Actinomycin D (ActD, catalog number: A4448, APExBIO, Shanghai, China) was added to each well at a final concentration of 10 μg/mL. Cells were then collected at designated time points following treatment over varying time intervals. Subsequently, total RNA was isolated, and the relative expression levels of *STAT5B* were evaluated using RT-qPCR.

### **2.19. MethylTarget Sequencing** 

The MethylTarget™ sequencing was performed by E-GENE Tech Co., Ltd., located in Shenzhen, China [18]. The EZ DNA Methylation-Gold™ Kit (catalog number: DP215, TIANGEN Biotech, Beijing, China) was utilized to convert all unmethylated cytosines into uracils. Any samples that showed a bisulfite conversion rate of less than 98% were excluded from further analysis. Following the amplification, separation, and purification of the target CpG regions, a CpG island methylation assay was conducted using an Illumina Hiseq/Miseq 2000 (San Diego, CA, USA), according to the manufacturer’s instructions.

### **2.20. DNA Extraction and Bisulfite Conversion** 

Genomic DNA was extracted using the Biospin Genomic DNA Extraction Kit from Sangon Biotech (catalog number: B8251, Shanghai, China), according to the manufacturer’s instructions. For the bisulfite conversion process, 500 ng of the purified DNA was treated with the EZ DNA Methylation-Gold™ Kit from TIANGEN Biotech (catalog number: DP215, Beijing, China). The procedure involved DNA denaturation at 98 °C for 10 min, followed by bisulfite conversion at 64 °C for 2.5 h. After conversion, the DNA was purified using spin columns and then eluted in 20 μL of Tris-EDTA buffer (pH 8.0) and stored at –20 °C until it was needed for further analysis.

### **2.21. Methylation-Specific PCR Assay** 

Methylation-specific PCR (MSP) was conducted to assess the methylation status using the MSP Kit from TIANGEN Biotech (catalog number: EM101, Beijing, China), which contains primers specifically designed for bisulfite-converted sequences. The reaction mixture was prepared in a total volume of 20 μL, comprising less than 500 ng of template DNA, 1 μL each of the forward and reverse primers at a concentration of 10 μM, 1.6 μL of dNTPs at 2.5 mM, 1 unit of MSP DNA polymerase, 2 μL of 10× MSP buffer, and nuclease-free water to achieve the final volume. The thermocycling protocol began with an initial denaturation step at 95 °C for 5 min, followed by 35 cycles of amplification at 94 °C for 20 s, 60 °C for 30 s, and 72 °C for 20 s. A final extension step was performed at 72 °C for 5 min. The PCR products (approximately 400 bp) were subsequently analyzed by 2% agarose gel electrophoresis and visualized under UV light.

### **2.22. Statistical Analysis** 

Data are presented as means ± standard error of the mean (SEM). To evaluate statistical differences between the two groups, Student’s *t*-test was utilized. For analyses involving multiple groups, one-way ANOVA was performed, followed by either the least significant difference (LSD) test or Dunnett’s T3 post hoc test. All statistical evaluations were conducted using SPSS version 26. Data visualization was executed with GraphPad Prism version 8.0.2. Correlations were assessed using Pearson correlation analysis, with a *p*-value of less than 0.05 considered statistically significant.

## 3. Results

### **3.1. Oligodendrocyte-Driven Myelin Maturation Defects Identified by snRNA-Seq in the MPTP-Induced Mouse Model** 

**Figure 1:**
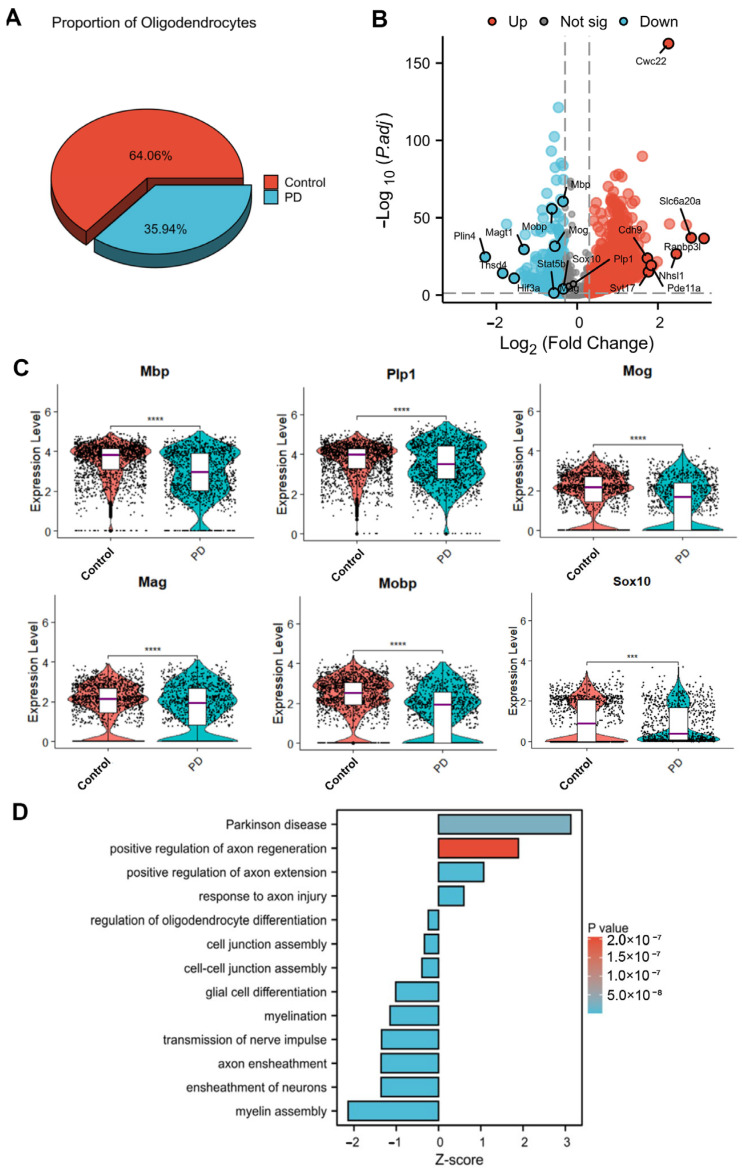
Characterization of the single-nucleus transcriptome profile of oligodendrocytes in the PD model. (**A**) The proportion of oligodendrocytes in the control group and MPTP-induced model (PD) group in the total oligodendrocyte cluster. (**B**) Volcano plot of DEGs in oligodendrocytes. (**C**) Violin plots showing the changes in the expression of myelin-related genes (***, *p* < 0.001; ****, *p* < 0.0001). (**D**) Bar plots of GO and KEGG term enrichment for DEGs in oligodendrocytes.

To investigate the role of oligodendrocytes in PD, we analyzed snRNA-seq data from the SN of MPTP (PD) and control mice (Supplementary Figure S1A) [19]. Our analysis revealed a significant reduction in the oligodendrocyte proportion in the PD group (Figure 1A). To characterize transcriptional alterations in oligodendrocytes under PD conditions, we performed differential gene expression analysis (*p* < 0.05, |log2FC| > 0.2; Figure 1B). Notably, key myelin-related genes (*Sox10*, *Plp1*, *Mbp*, *Mog*, *Mag*, *Mobp*) showed significant downregulation in PD (Figure 1C), suggesting a compromised myelination capacity. Integrated pathway analysis using clusterProfiler (v4.0.3) (for Gene Ontology [GO] and Kyoto Encyclopedia of Genes and Genomes [KEGG] pathway enrichment) with Log2FC showed that PD oligodendrocytes exhibited impaired core functions, including myelination and axon ensheathment, accompanied by the dysregulated activation of pathways related to Parkinson’s disease progression, oligodendrocyte differentiation, and axonal injury response (Figure 1D). These findings collectively demonstrate substantial transcriptomic alterations in PD oligodendrocytes, characterized by suppressed myelin maintenance functions and the activation of pathogenic pathways associated with disease progression.

**Figure 2:**
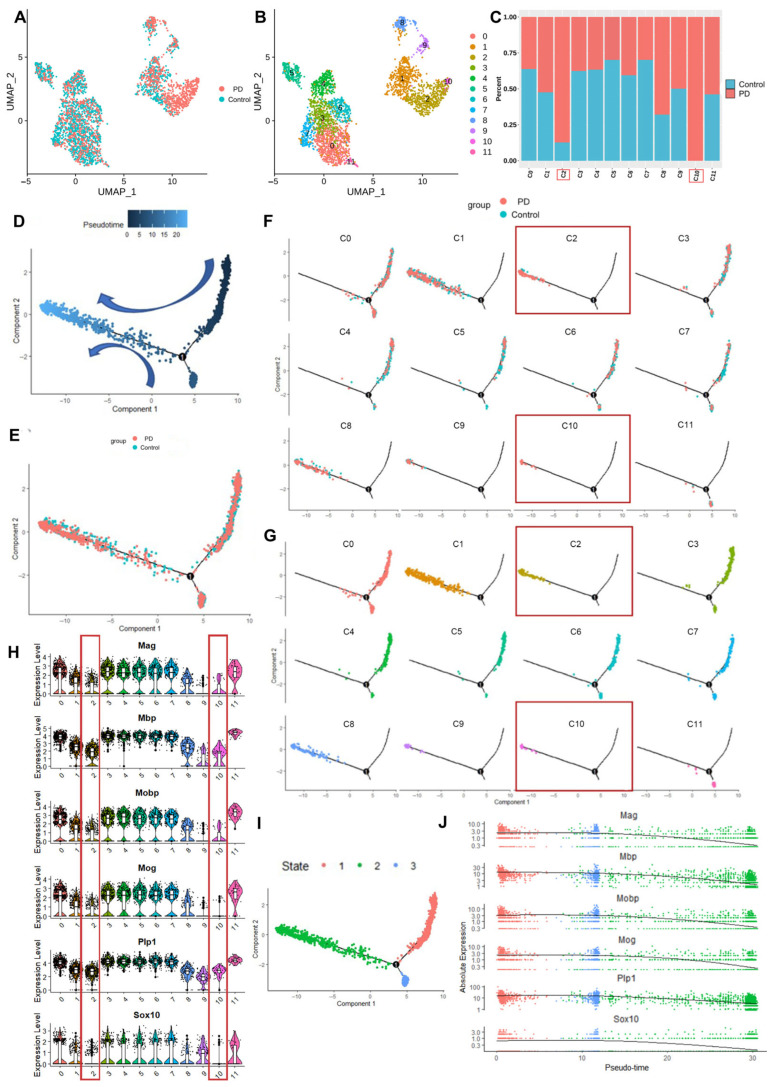
Molecular characteristics of oligodendrocyte maturation impairment. (**A**) UMAP embedding of oligodendrocyte nuclei, colored by group (control vs. PD). (**B**) UMAP embedding of oligodendrocyte nuclei, colored by cluster (11 subclusters). (**C**) Proportional representation of cells in the 11 subclusters, highlighting that clusters C2 and C10 primarily consist of oligodendrocytes from the PD group. (**D**) Monocle2 pseudotime analysis of the 11 clusters. (**E**,**F**) Time trajectory plots showing disease progression over time in control and PD groups. (**G**) Time trajectory plot of subclusters, indicating that oligodendrocyte clusters C2 and C10 are at the end of the differentiation trajectory. The C2 and C10 are marked in red rectangular frames. (**H**) Violin plot of the 11 subclusters, showing the significant downregulation of myelin-related genes in clusters C2 and C10. (**I**) Division of the time trajectory into three states. (**J**) Scatter plot demonstrating the dynamic expression of myelin-related genes across the three states.

To elucidate the cellular heterogeneity and functional states of oligodendrocytes in PD, we conducted a subclustering analysis of the oligodendrocyte population. Uniform manifold approximation and projection (UMAP) visualization revealed distinct spatial segregation between control and PD-derived oligodendrocytes (Figure 2A). Further reclustering identified 12 transcriptionally distinct subpopulations (Figure 2B), among which subclusters C2 and C10 showed predominant enrichment in PD samples (Figure 2C), suggesting their potential association with pathological oligodendrocyte states during PD progression.

To characterize subclusters C2 and C10 and their potential involvement in PD pathogenesis, we conducted a pseudotemporal trajectory analysis using Monocle2 (Figure 2D). The reconstructed differentiation trajectories, stratified by experimental condition (Figure 2E,F) and subcluster identity (Figure 2G), revealed the progressive accumulation of PD-derived oligodendrocytes along the pseudotime axis, with subclusters C2 and C10 predominantly localized to the terminal differentiation state. Notably, the expression analysis of myelin-related genes (*Sox10*, *Plp1*, *Mbp*, *Mog*, *Mag*, *Mobp*) across all 12 subclusters demonstrated significant downregulation in C2 and C10 (Figure 2H), confirming their association with functionally impaired oligodendrocyte states characterized by a deficient myelination capacity in PD.

An analysis of the oligodendrocyte differentiation pseudotime trajectories revealed three distinct states, enabling the characterization of myelin-related gene expression patterns (Figure 2I). We observed the progressive downregulation of myelin-related genes (*Sox10*, *Plp1*, *Mbp*, *Mog*, *Mag*, *Mobp*) across these developmental states (Figure 2J), suggesting that PD pathogenesis disrupts normal oligodendrocyte maturation and ultimately compromises myelin sheath integrity.

### **3.2. Myelin Defects in the MPTP-Induced Mouse Model** 

We established a subacute PD model via the intraperitoneal injection of MPTP. Immunohistochemical staining revealed significantly lower tyrosine hydroxylase (TH) expression in the SN of the PD group compared to the control group (Supplementary Figure S1B,C). Behavioral assessments, including the rotarod test (Supplementary Figure S1D), climbing pole test (Supplementary Figure S1E,F), and gait analysis (Supplementary Figure S1G–I), demonstrated motor dysfunction in PD mice, confirming the successful establishment of the model.

**Figure 3:**
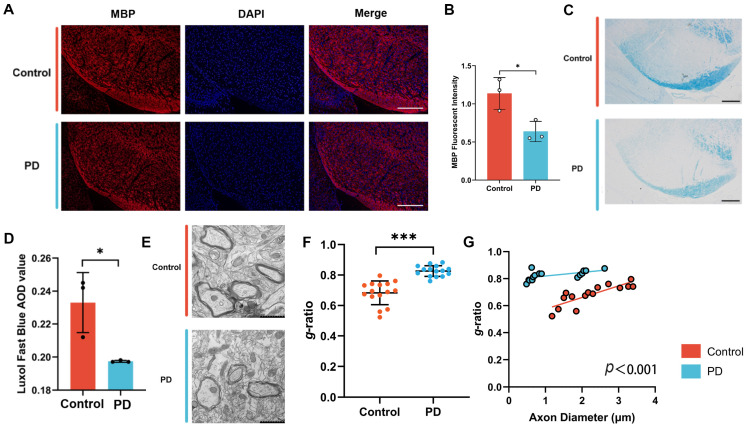
Myelin in the SN was injured in the MPTP-induced mouse model. (**A**,**B**) Immunofluorescence staining reveals a significant reduction in MBP expression levels in the SN of the MPTP-induced mouse model. The results are expressed as the mean ± SD (10× scale bars = 200 μm). Data are presented from male (n = 3) mice, aged 8 weeks. (**C**,**D**) Luxol Fast Blue (LFB) staining shows a decrease in myelin density in the SN of the MPTP-induced mouse model. The results are expressed as the mean ± SD (10× scale bars = 200 μm). Data are presented from male (n = 3) mice, aged 8 weeks. (**E**,**F**) Transmission electron microscopy (TEM) showed that axonal myelin sheaths were loose, with a reduced thickness and significantly increased g-ratio, in the SN of the MPTP-induced mouse model. The results are expressed as the mean ± SD (5 randomly selected myelinated axons per SN field; 20.0 k× scale bars = 1 μm). Data are presented from male (n = 3) mice, aged 8 weeks. (**G**) Scatter plot illustrating the individual *g*-ratio values and the distribution of axonal sizes (*, *p* < 0.05; ***, *p* < 0.001).

We first evaluated myelin integrity via the immunofluorescence staining of MBP, a core structural protein specifically expressed by oligodendrocytes and essential for myelin sheath formation and integrity [20]. The results showed significantly reduced MBP expression in the SN of PD mice compared to controls (Figure 3A,B). To further characterize myelin damage, we performed Luxol Fast Blue (LFB) staining and quantified the myelin density using average optical density (AOD) measurements. Histological examination revealed myelin structural disruption in the PD group, characterized by a reduced tissue density, paler LFB staining, and significantly lower AOD values compared to controls (Figure 3C,D). Transmission electron microscopy (TEM) further confirmed myelin abnormalities in the SN, including loose myelin sheaths, irregular thicknesses, and a significantly increased *g*-ratio in PD mice compared to controls (Figure 3E,F). Together, these findings consistently indicate impaired myelin maturation in the SN and compromised myelin integrity in the MPTP-induced mouse model.

### **3.3. Oligodendrocyte STAT5B Was a Key Regulator of Myelin Injury in the MPTP-Induced Mouse Model** 

**Figure 4:**
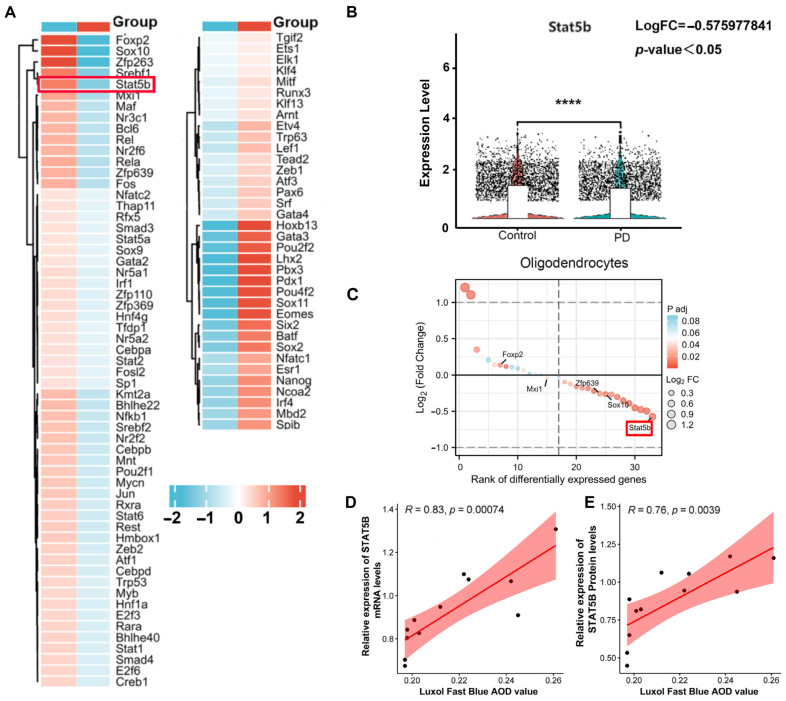
Discovery and validation of STAT5B as a key regulatory factor. (**A**) DoRothEA analysis identifies 32 TFs with significantly altered activity in oligodendrocytes in the PD group (12 activated, 20 downregulated). (**B**) Differential ranking of TFs reveals significant downregulation of *STAT5B* mRNA in snRNA-seq results (****, *p* < 0.0001). (**C**) Violin plot confirms significant decrease in STAT5B expression in oligodendrocytes from the PD group compared to the control group. (**D**,**E**) LFB staining shows decreased myelin density correlating with STAT5B mRNA (r = 0.83, *p* < 0.00074) and protein (r = 0.76, *p* < 0.0039) expression in the SN of mice.

To identify key transcriptional regulators underlying oligodendrocyte dysfunction in PD, we performed TF activity analysis using DoRothEA. This approach revealed 96 TFs with significantly altered activity (Figure 4A), including 37 with increased activity and 59 with decreased activity in PD oligodendrocytes. Consistent with the DoRothEA analysis, we identified STAT5B as the most significantly downregulated TF in the MPTP-induced mouse model (Figure 4B). Specifically, we observed a significant decrease in *STAT5B* expression (log2FC = −0.576, *p* < 0.05) in oligodendrocytes from the PD group compared to the controls (Figure 4C). We performed Pearson correlation analyses to evaluate the relationship between the myelin density (quantified by AOD values from LFB staining) and STAT5B expression. The results revealed that a reduced myelin density strongly correlated with STAT5B mRNA (r = 0.83, *p* = 0.00074; Figure 4D) and protein (r = 0.76, *p* = 0.0039; Figure 4E), suggesting a potential role for STAT5B in maintaining myelin integrity.

**Figure 5:**
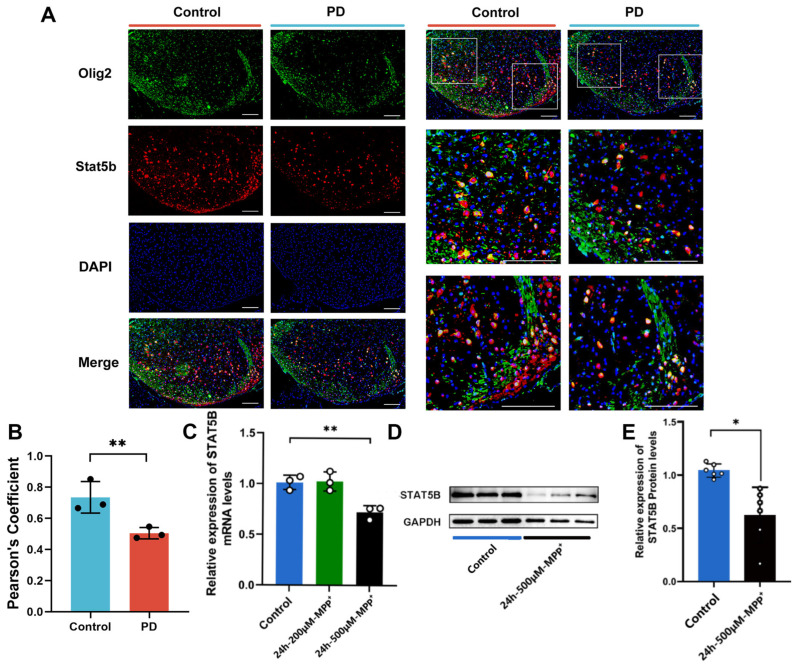
The expression of STAT5B was significantly reduced in the PD model. (**A**) Representative image of immunofluorescence staining in the SN of the MPTP-induced mouse model (scale bars = 100 μm). (**B**) Quantification of co-localization using Pearson’s correlation coefficient (PCC). The y-axis represents the PCC, which quantifies the degree of spatial overlap between the STAT5B and Olig2 signals. PCC values range from −1 to +1, where +1 indicates perfect co-localization, 0 indicates no correlation (random distribution), and -1 indicates perfect segregation. Each data point represents the PCC calculated from individual image fields. The data are presented as the means ± SEMs of 3 independent experiments. Data are presented from male (n = 3) mice, aged 8 weeks. (**C**) qRT-PCR analysis of *STAT5B* mRNA expression in MO3.13 cells treated with 200 and 500 μM MPP^+^ for 24 h. The data are presented as the means ± SEMs of 3 independent experiments. (**D**,**E**) Western blot analysis of STAT5B protein expression in MO3.13 cells treated with 500 μM MPP^+^ for 24 h. The data are presented as the means ± SEMs of 6 independent experiments (*, *p* < 0.05; **, *p* < 0.01).

We further validated this finding using multiplex immunofluorescence staining, which revealed a significant reduction in STAT5B protein levels within Olig2+ oligodendrocytes in the PD group (Figure 5A,B). To investigate the pathological state of oligodendrocytes in PD, we treated MO3.13 cells with MPP^+^, a neurotoxin that recapitulates key PD-associated cellular damage. CCK-8 assays identified optimal treatment conditions (0, 50, 100, 200, and 500 μM MPP^+^, 4–48 h), revealing significant viability losses at 200 and 500 μM after 24 h (Supplementary Figure S2A). The qRT-PCR and Western blot showed that STAT5B mRNA and protein were significantly downregulated at 500 μM MPP^+^ (24 h) versus controls (Figure 5C–E). The concordant downregulation of oligodendrocytic STAT5B in both PD models (in vivo and in vitro) suggests its potential role in myelin impairment and PD pathogenesis.

### **3.4. STAT5B Overexpression in Oligodendrocytes Prevents Myelin Damage in the MPTP-Induced Mouse Model** 

**Figure 6:**
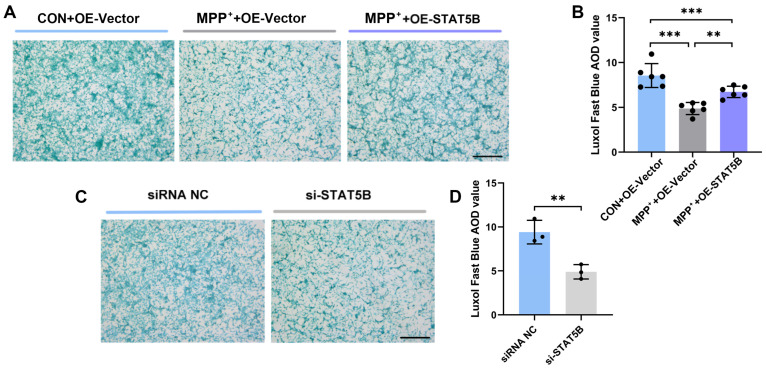
LFB staining of myelin in co-cultures of differentiated MO3.13 cells and SH-SY5Y neuronally differentiated cells. (**A**,**B**) LFB staining of *STAT5B* overexpression in MPP^+^-treated MO3.13 cells. (**C**,**D**) LFB staining of *STAT5B* knockdown in MO3.13 cells. The results are expressed as the mean ± SD (n = 3; 10× scale bars = 200 μm; **, *p* < 0.01; ***, *p* < 0.001).

To investigate the protective role of STAT5B regarding myelin integrity, we overexpressed *STAT5B* in MPP^+^-treated MO3.13 cells. Successful *STAT5B* overexpression was confirmed through transfection efficiency assays (Supplementary Figure S2B–D). Then, we established a co-culture system of MO3.13 cells with SH-SY5Y neuronally differentiated cells. Myelin damage was quantified using LFB staining. Compared to normal co-culture controls (CON+OE-Vector), the MPP^+^-treated co-culture controls (MPP^+^+OE-Vector) showed significantly reduced AOD values. *STAT5B* overexpression (MPP^+^+OE-STAT5B) significantly attenuated this damage, as demonstrated by increased AOD values (Figure 6A,B), indicating that the overexpression of *STAT5B* alleviated MPP^+^-induced myelin damage.

Having demonstrated the protective effects of *STAT5B* through overexpression (Figure 6A,B), we next examined the consequences of *STAT5B* depletion. Transfection with two independent *STAT5B*-targeting siRNAs (si-STAT5B-1/2) effectively reduced both the mRNA and protein levels (Supplementary Figure S2E–G). In the MO3.13/SH-SY5Y co-culture system, *STAT5B* knockdown significantly decreased the AOD values of LFB staining versus controls (Figure 6C,D), demonstrating that *STAT5B* deficiency exacerbates myelin damage in this PD model system.

**Figure 7:**
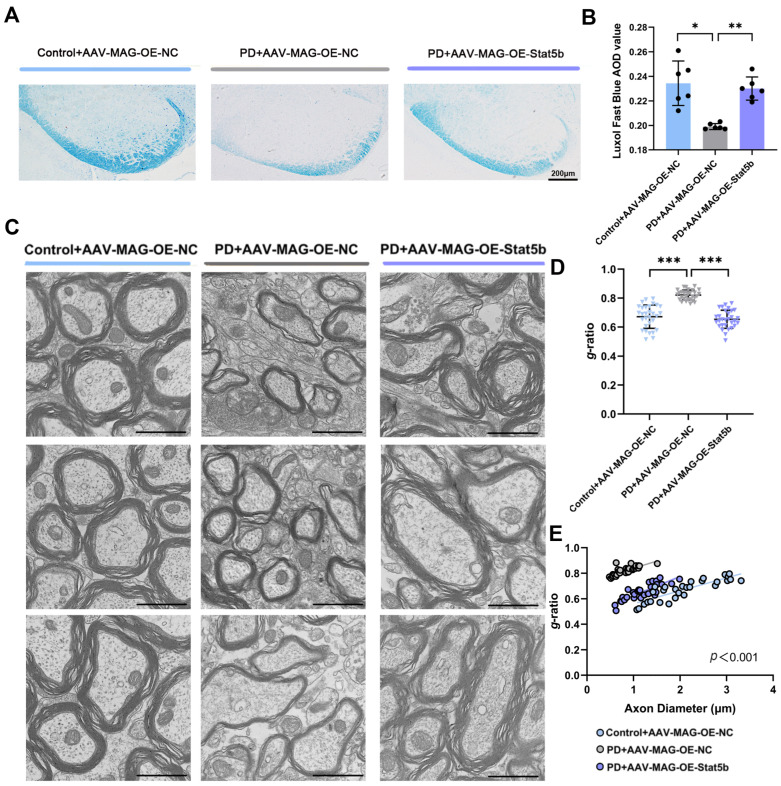
Overexpression of oligodendrocyte *STAT5B* improved myelin damage in MPTP-induced mice. (**A**,**B**) LFB staining showed that overexpression of oligodendrocyte *STAT5B* increased myelin density in MPTP-induced mice. The results are expressed as the mean ± SD (10× scale bars = 200 μm). Data are presented from male (n = 6) mice, aged 8 weeks. (**C**,**D**) TEM analysis revealed that oligodendrocyte *STAT5B* overexpression increased axonal myelin thickness and significantly reduced g-ratio in MPTP-treated mice. The results are expressed as the mean ± SD (5 randomly selected myelinated axons per SN field; 20.0 k× scale bars = 1 μm). (**E**) Scatter plot illustrating the individual *g*-ratio values and the distribution of axonal sizes. Data are presented from male (n = 3) mice, aged 8 weeks (*, *p* < 0.05; **, *p* < 0.01; ***, *p* < 0.01).

We next validated these effects in vivo using oligodendrocyte-targeted AAV-MAG-OE-STAT5B, which was constructed under the control of the myelin-associated glycoprotein (MAG) promoter. Successful SN delivery was confirmed by immunofluorescence (Supplementary Figure S3A). Both the qRT-PCR and Western blot analysis (Supplementary Figure S3B–D) of the SN demonstrated significantly elevated *STAT5B* expression levels in the PD+AAV-MAG-OE-STAT5B group compared to the PD model control group (PD+AAV-MAG-OE-NC). LFB staining revealed significantly higher AOD values in *STAT5B*-overexpressing PD mice (PD+AAV-MAG-OE-STAT5B) compared to the PD+AAV-MAG-OE-NC group (Figure 7A,B), indicating the restoration of myelin integrity following *STAT5B* overexpression. TEM ultrastructural analysis revealed that the PD+AAV-MAG-OE-STAT5B group significantly reduced the elevated g-ratio observed in the PD+AAV-MAG-OE-NC group (Figure 7C,D), further confirming STAT5B’s protective role against MPTP-induced myelin damage in the SN.

### **3.5. Oligodendrocyte STAT5B Prevents Dopaminergic Neuronal Degeneration and Behavioral Impairment by Improving Myelin Function** 

**Figure 8:**
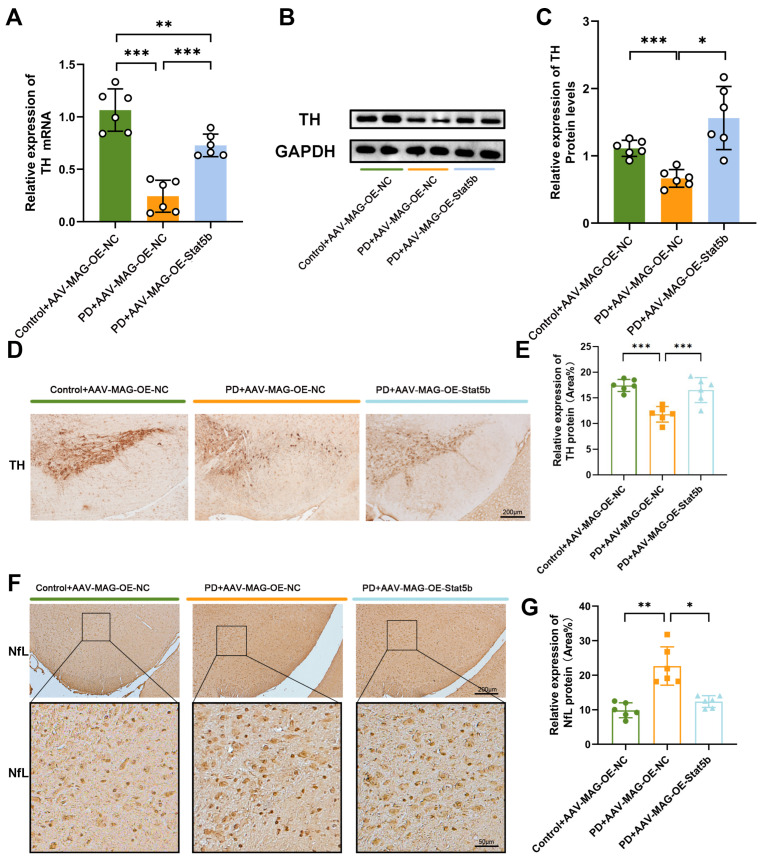
Overexpression of oligodendrocyte *STAT5B* ameliorated dopamine neuronal damage in MPTP-treated mice. (**A**) qRT-PCR analysis of TH mRNA expression in the mouse SN. (**B**) Western blot analysis and (**C**) quantification of TH protein levels in the mouse SN. (**D**,**E**) Immunohistochemical staining of TH protein in the mouse SN. (**F**,**G**) Immunohistochemical staining of NfL protein in the mouse SN. The results are expressed as the mean ± SD (10× scale bars = 200 μm; 40× scale bars = 50 μm; *, *p* < 0.05; **, *p* < 0.01; ***, *p* < 0.001). Data are presented from male (n = 6) mice, aged 8 weeks.

We next examined whether STAT5B-mediated myelin protection influenced dopaminergic neuron survival. The PD+AAV-MAG-OE-STAT5B group showed significantly increased TH mRNA and protein levels compared to the PD+AAV-MAG-OE-NC group (Figure 8A–C), with immunohistochemistry confirming the enhanced TH expression (Figure 8D,E). Furthermore, immunohistochemical analysis revealed significantly increased neurofilament light chain (NfL) protein expression in the PD+AAV-MAG-OE-NC group compared to the Control+AAV-MAG-OE-NC group. *STAT5B*-overexpressing PD mice reduced the elevated NfL levels in the MPTP-induced mouse model (Figure 8F,G). These findings demonstrate that oligodendrocyte-specific *STAT5B* overexpression mitigates dopaminergic neuron damage in the MPTP-induced PD mouse model.

**Figure 9:**
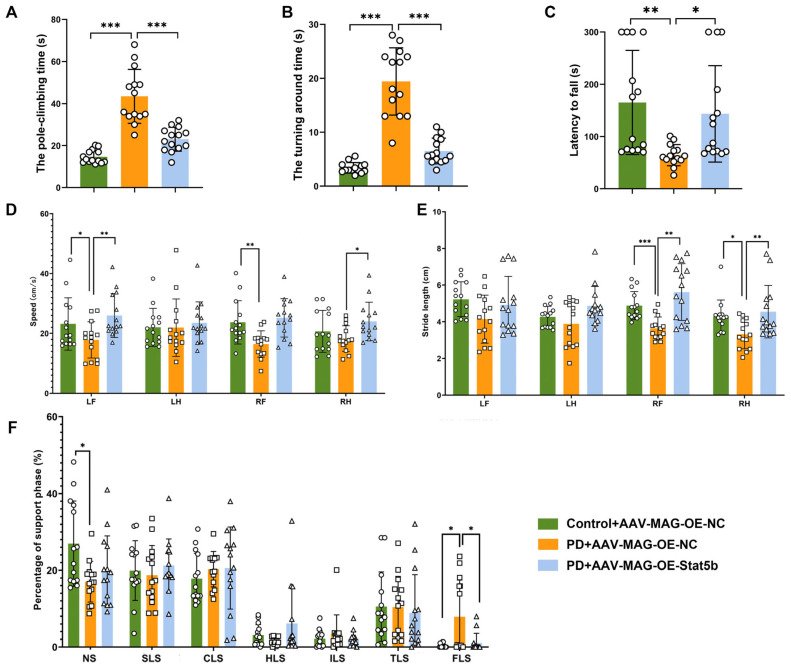
Overexpression of oligodendrocyte *STAT5B* improved motor function in MPTP-treated mice. (**A**,**B**) Pole test results showing total time and turn time. (**C**) Rotarod test results showing fall latency. (**D**–**F**) Gait analysis results showing movement speed, stride length, and support time. NS: no support, SLS: single-leg support, CLS: contralateral limb support, HLS: homologous limb support, ILS: ipsilateral limb support, TLS: three-limb support, FLS: four-limb support. The results are expressed as the mean ± SD (*, *p* < 0.05; **, *p* < 0.01; ***, *p* < 0.001). Data are presented from male (n = 14) mice, aged 8 weeks.

Finally, we performed behavioral tests to assess its effects on motor dysfunction in the MPTP-induced mouse model. The pole climbing test indicated that the PD+AAV-MAG-OE-STAT5B group significantly decreased both the total climbing time and turning around time compared to the PD+AAV-MAG-OE-NC group (Figure 9A,B). The rotarod test revealed that the fall latency was significantly greater in the PD+AAV-MAG-OE-STAT5B group than in the PD+AAV-MAG-OE-NC group (Figure 9C). Gait analysis showed that the PD+AAV-MAG-OE-NC group, compared to the Control+AAV-MAG-OE-NC group, exhibited significant gait impairments, characterized by a reduced movement speed and stride length and increased four-limb support time (Figure 9D–F). The PD+AAV-MAG-OE-STAT5B group significantly ameliorated these deficits, increasing the movement speed and stride length while decreasing the support time (Figure 9D–F). These results collectively demonstrate that *STAT5B* overexpression effectively ameliorates motor impairments in the MPTP-induced PD mouse model.

### **3.6. Oligodendrocyte STAT5B Improves Myelin Injury via Transcriptional Activation of MBP** 

**Figure 10:**
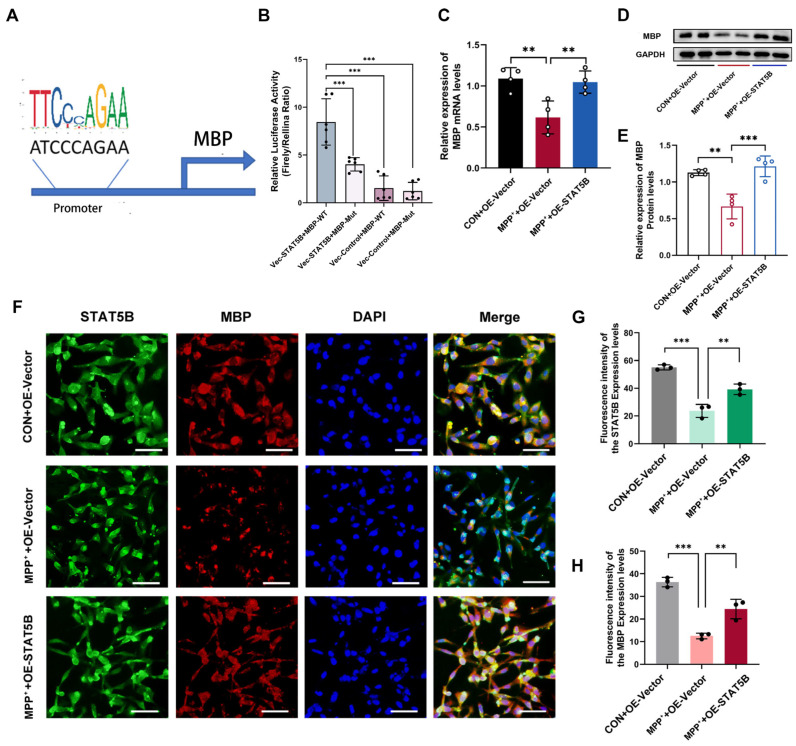
*STAT5B* overexpression promoted *MBP* expression and reduced myelin injury. (**A**,**B**) Luciferase reporter assay showing the binding efficiency of *STAT5B* to the *MBP* promoter region (n = 6). (**C**–**H**) In MO3.13 cells overexpressing *STAT5B*: (**C**) qRT-PCR and (**D**,**E**) Western blot analysis of MBP expression (n = 4). (**F**–**H**) Immunofluorescently stained *STAT5B* and *MBP* expression (n = 3, scale bars = 50 μm). The results are expressed as the mean ± SD (**, *p* < 0.01; ***, *p* < 0.001).

Given our experimental evidence for the critical role of STAT5B in oligodendrocyte myelin maintenance, we investigated its direct transcriptional regulation of myelin-related genes. Bioinformatics analysis (hTFtarget and JASPAR) identified a high-affinity *STAT5B* binding site in the *MBP* promoter (Figure 10A). Luciferase reporter assays confirmed STAT5B-mediated transcriptional activation. The relative luciferase activity was increased by *STAT5B* overexpression in cells transfected with the wild-type *MBP* promoter (MBP-WT), while no significant difference was observed in the mutant constructs (MBP-Mut) (Figure 10B).

**Figure 11:**
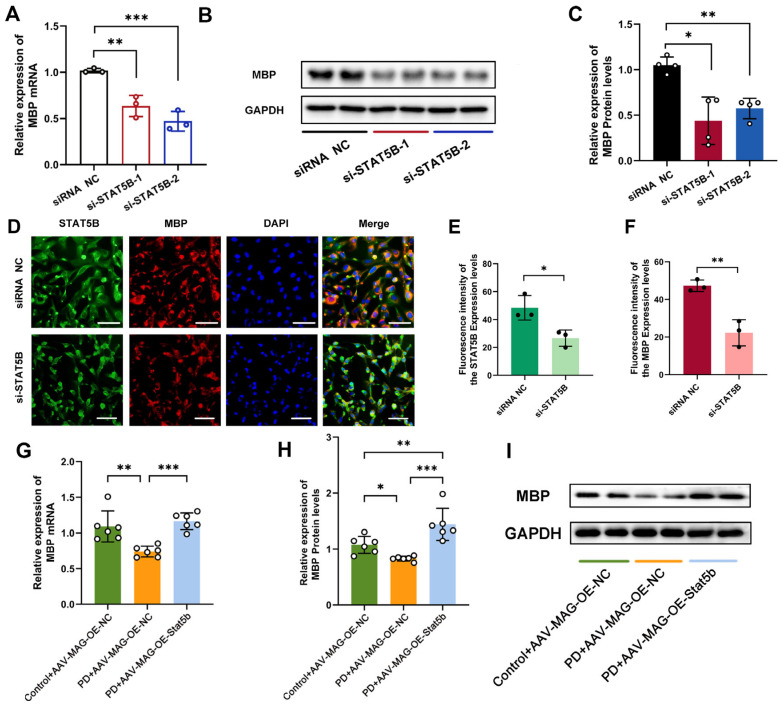
Knockdown of *STAT5B* decreases *MBP* expression and aggravates myelin damage. (**A**–**F**) In *STAT5B*-knockdown MO3.13 cells: (**A**) qRT-PCR analysis of *MBP* mRNA expression (n = 4), (**B**,**C**) Western blot analysis of MBP protein expression (n = 4), and (**D**–**F**) immunofluorescently stained STAT5B and MBP expression (n = 3, scale bars = 50 μm). (**G**) qRT-PCR analysis of *MBP* mRNA expression in the mouse SN. (**H**,**I**) Western blot analysis and quantification of MBP protein levels in the mouse SN. The results are expressed as the mean ± SD (*, *p* < 0.05; **, *p* < 0.01; ***, *p* < 0.001). Data are presented from male (n = 6) mice, aged 8 weeks.

Subsequently, we analyzed the MBP expression levels in MPP^+^-treated MO3.13 cells. Western blot and qRT-PCR analyses demonstrated significantly reduced MBP expression in the MPP^+^+OE-Vector group compared to the CON+OE-Vector group. *STAT5B* overexpression effectively rescued MBP expression (Figure 10C–E). These findings were further corroborated by immunofluorescence staining, which showed a significantly stronger MBP fluorescence intensity in the MPP^+^+OE-STAT5B group compared to the MPP^+^+OE-Vector group (Figure 10F–H). In contrast, the knockdown *STAT5B* (si-STAT5B group) significantly reduced MBP expression compared to the siRNA NC group (Figure 11A–F), further confirming that STAT5B is essential in improving MBP expression.

To validate these findings in vivo, we examined MBP expression in the SN of the MPTP-induced mouse model with oligodendrocyte-specific *STAT5B* overexpression. Compared to the Control+AAV-MAG-OE-NC group, the MBP mRNA and protein levels were significantly decreased in the PD+AAV-MAG-OE-NC group. Importantly, *STAT5B* overexpression (PD+AAV-MAG-OE-STAT5B) effectively restored MBP expression (Figure 11G–I). These results confirm that STAT5B transcriptionally regulates *MBP* expression to maintain proper oligodendrocyte myelination function.

### **3.7. DNMT3A Reduces STAT5B Expression Through DNA Methylation** 

**Figure 12:**
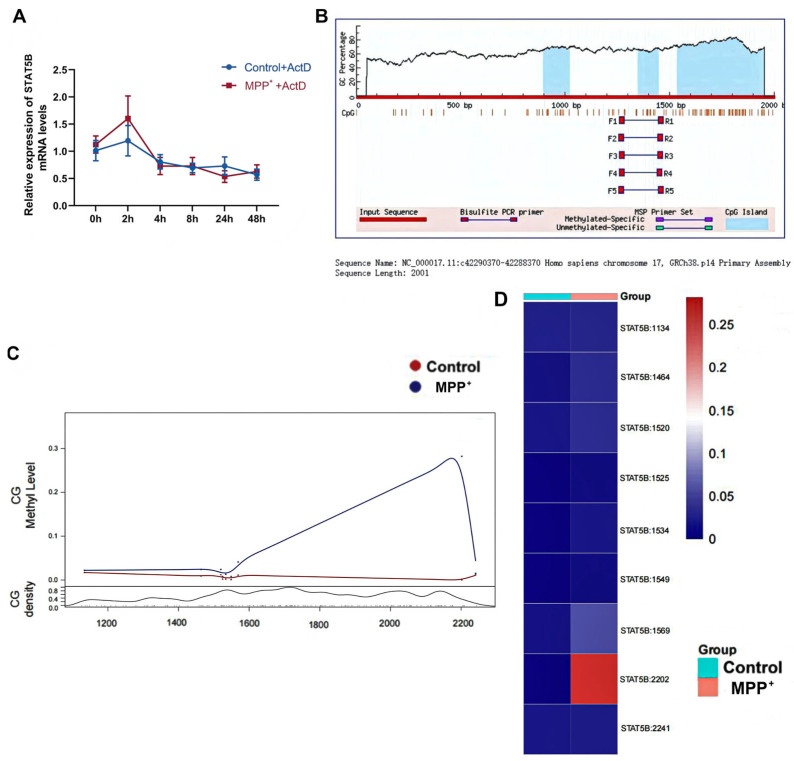
*STAT5B* mRNA stability and promoter methylation study. (**A**) Actinomycin D transcription inhibition assay to detect *STAT5B* mRNA stability. Line chart showing *STAT5B* mRNA expression at different time points after treatment with actinomycin D. The results are expressed as the mean ± SD (n = 6). (**B**) The MethPrimer website predicts that there are extensive CpG islands in the promoter region of *STAT5B* (light blue areas are CpG island regions). (**C**,**D**) Top 10 CpG sites with significantly increased methylation in the PD group compared to the control group, as determined by MethylTarget sequencing: (**C**) MethylTarget sequencing scatter plot—Y-axis: “CG methyl level”, “CG density”; X-axis: methylation site positions. (**D**) MethylTarget sequencing heatmap—Y-axis: methylation sites; X-axis: groups.

**Figure 13:**
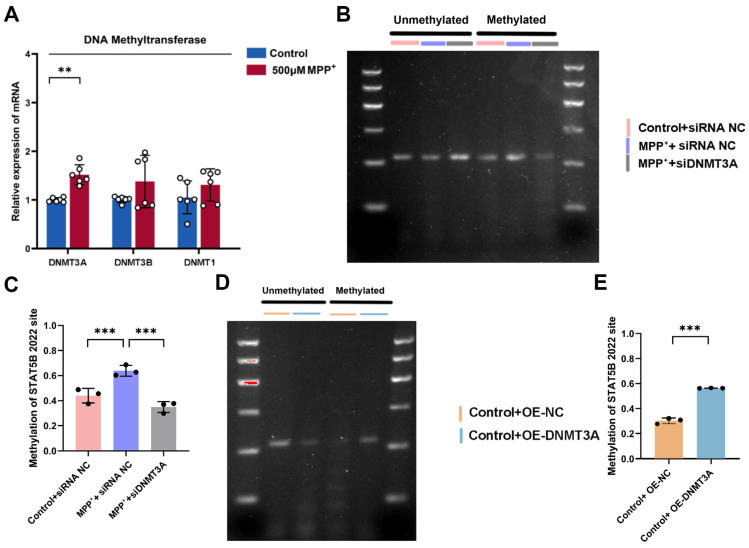
Effects of DNMT3A on methylation levels in the *STAT5B* promoter region. (**A**) mRNA expression of DNA methylation-related genes assessed by qRT-PCR (n = 6, **, *p* < 0.01; ***, *p* < 0.001). (**B**,**C**) Methylation-specific PCR (MSP) analysis of the *STAT5B* promoter region at site 2202 in *DNMT3A*-knockdown and MPP^+^-induced MO3.13 cells. (**D**,**E**) MSP analysis of the *STAT5B* promoter region at site 2202 in *DNMT3A*-overexpression MO3.13 cells.

To explore STAT5B’s upstream regulation in PD, we examined whether MPP^+^-induced neurotoxicity altered *STAT5B* expression via mRNA stability. ActD chase assays showed no change in *STAT5B* mRNA stability after MPP^+^ treatment (Figure 12A), indicating that *STAT5B* dysregulation occurs through mechanisms other than post-transcriptional mRNA degradation. MethPrimer analysis revealed dense CpG islands in the *STAT5B* promoter (Figure 12B), suggesting methylation-dependent regulation. MethylTarget bisulfite sequencing showed significantly increased methylation at CpG site 2202 in PD models (Figure 12C,D), implicating this locus in MPP^+^-induced *STAT5B* downregulation in MO3.13 cells. qRT-PCR analysis revealed the MPP^+^-induced upregulation of *DNMT3A* (Figure 13A), the de novo methyltransferase responsible for establishing new methylation patterns [21].

We investigated the role of DNMT3A in *STAT5B* promoter methylation at CpG site 2202 using methylation-specific PCR (MSP). Compared to controls (Control+siRNA-NC), the MPP^+^+siRNA-NC group showed significantly increased methylation at CpG site 2202 in the *STAT5B* promoter, which was significantly reduced by *DNMT3A* knockdown (MPP^+^+siDNMT3A) (Figure 13B,C). *DNMT3A* overexpression (Control+OE-DNMT3A) significantly enhanced methylation at CpG site 2202 in the *STAT5B* promoter compared to controls (Control+OE-NC) (Figure 13D,E), demonstrating that the DNMT3A-mediated methylation of this locus contributes to *STAT5B* transcriptional repression.

**Figure 14:**
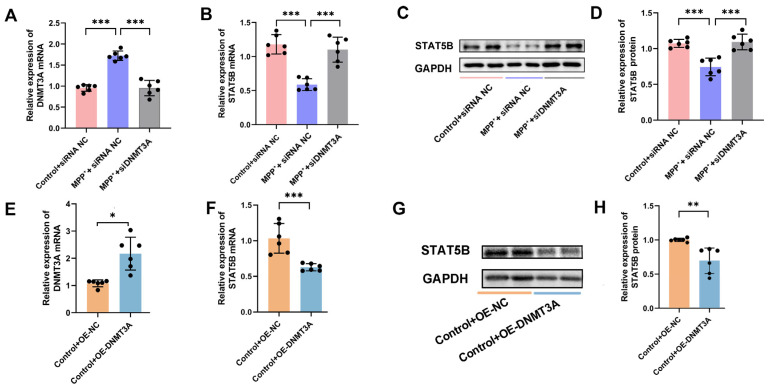
*DNMT3A* knockdown and overexpression affect *STAT5B* expression. (**A**–**D**) In *DNMT3A*-knockdown MO3.13 cells: (**A**–**D**) qRT-PCR and (**C**,**D**) Western blot analysis of STAT5B expression (n = 6). (**E**–**H**) In MO3.13 cells with *DNMT3A* overexpression or *STAT5B* co-overexpression with *DNMT3A*: (**E**,**F**) qRT-PCR analysis of *DNMT3A* and *STAT5B* mRNA and (**G**,**H**) Western blot analysis of STAT5B protein expression (n = 6). The results are expressed as the mean ± SD (*, *p* < 0.05; **, *p* < 0.01; ***, *p* < 0.001).

We next examined the role of DNMT3A in regulating *STAT5B* expression through gain- and loss-of-function experiments. The MPP^+^+siDNMT3A group showed significantly reduced DNMT3A levels compared to the MPP^+^+siRNA-NC group, while increasing both STAT5B mRNA and protein expression (Figure 14A–D). Conversely, the Control+OE-DNMT3A group showed decreased *STAT5B* expression compared to the Control+OE-NC group (Figure 14E–H). These results establish DNMT3A as a key regulator of *STAT5B* expression.

**Figure 15:**
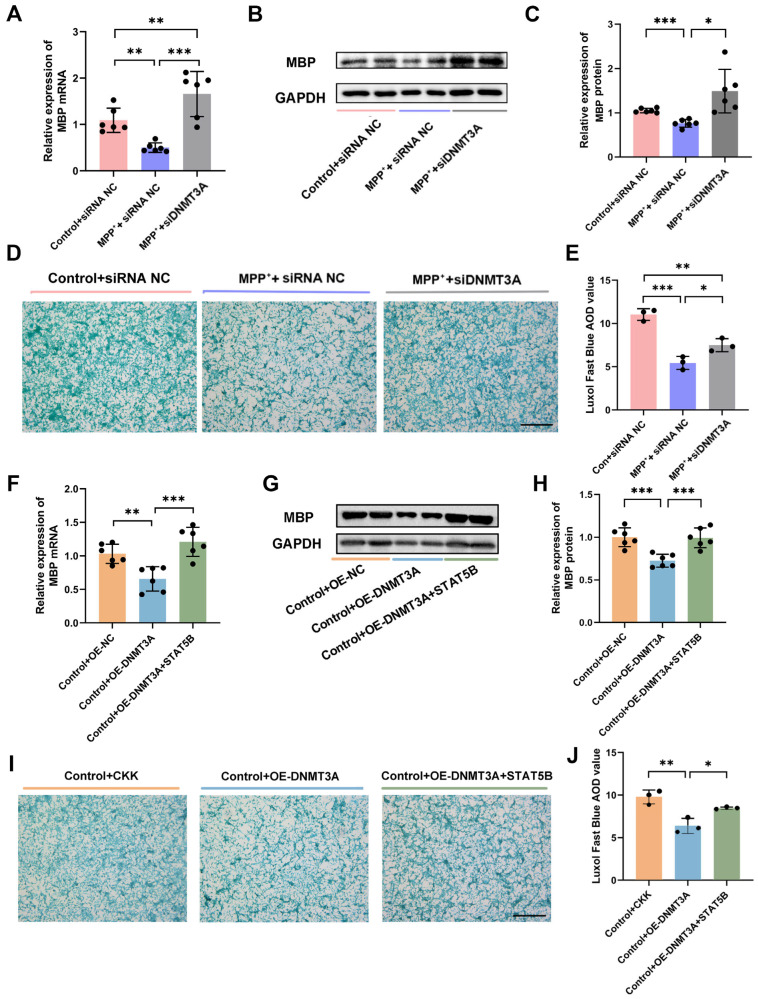
DNMT3A-mediated downregulation of *MBP* via STAT5B in oligodendrocytes. (**A**–**E**) In *DNMT3A*-knockdown MO3.13 cells: (**A**–**C**) qRT-PCR and Western blot analysis of MBP expression (n = 6). (**D**,**E**) LFB staining indicates improved myelin integrity in SH-SY5Y neuronally differentiated cells co-cultured with differentiated *DNMT3A*-knockdown MO3.13 cells (n = 3, scale bars = 200 μm). The results are expressed as the mean ± SD (*, *p* < 0.05; **, *p* < 0.01; ***, *p* < 0.001). (**F**–**J**) In MO3.13 cells with *DNMT3A* and *STAT5B* co-overexpression: (**F**–**H**) qRT-PCR and Western blot analysis of MBP expression (n = 6). (**I**,**J**) LFB staining shows improved myelin integrity in SH-SY5Y neuronally differentiated cells co-cultured with MO3.13 cells co-overexpressing *STAT5B* and *DNMT3A* (n = 3). The results are expressed as the mean ± SD (*, *p* < 0.05; **, *p* < 0.01; ***, *p* < 0.001).

Our studies demonstrated DNMT3A’s critical role in regulating both *MBP* expression and myelin integrity. *DNMT3A* knockdown (MPP^+^+siDNMT3A) significantly increased MBP expression at the mRNA and protein levels compared to the MPP^+^+siRNA-NC group (Figure 15A–C). This was further validated in co-culture systems, where *DNMT3A* knockdown improved myelin integrity, as evidenced by increased LFB staining (AOD values) versus MPP^+^+siRNA-NC (Figure 15D,E). Rescue experiments revealed that *DNMT3A* regulates myelination through STAT5B-dependent mechanisms. *DNMT3A* overexpression alone (Control+OE-DNMT3A) reduced MBP expression (Figure 15F–H), and *STAT5B* co-expression (Control+OE-DNMT3A+STAT5B) rescued this effect. Consistent results were observed in co-culture systems, where the Control+OE-DNMT3A+STAT5B group reversed DNMT3A-mediated reductions in the LFB staining intensity (AOD values; Figure 15I,J). These findings demonstrate DNMT3A’s role in myelin integrity through STAT5B epigenetic regulation.

## 4. Discussion

Our study significantly strengthens the understanding of oligodendrocytes’ involvement in PD pathogenesis. We identified a novel epigenetic pathway where DNMT3A-mediated hypermethylation represses *STAT5B* expression in oligodendrocytes, leading to the subsequent downregulation of *MBP* and myelin injury, ultimately exacerbating dopaminergic neuronal degeneration.

Oligodendrocytes are not only responsible for the formation and maintenance of the myelin sheath but are also actively involved in neuroprotective and repair processes. However, conventional research methods often fall short in fully capturing the heterogeneity of oligodendrocytes and their specific functions in PD. The advent of scRNA-seq has revolutionized this field, enabling the in-depth exploration of oligodendrocytes’ diversity and their role in PD at the single-cell level [22]. Our snRNA-seq analysis of the MPTP-induced mouse model revealed a decreased proportion of oligodendrocytes and the pervasive downregulation of myelin-related genes in the SN. Monocle2 pseudotime analysis further elucidated the dynamic transitions between oligodendrocyte subpopulations, revealing the accumulation of PD group cells at terminal differentiation states characterized by progressive myelin gene silencing. Furthermore, a scRNA-seq atlas of the human SN revealed a significant association between PD risk and oligodendrocyte-specific gene expression [23]. Another scRNA-seq analysis demonstrated the dysregulation of immune regulation and cytokine signaling pathways in oligodendrocytes and oligodendrocyte precursor cells (OPCs) from PD patients, consistent with the neuroinflammatory processes observed in PD [24]. Recent studies further support these findings, demonstrating that GPR37 upregulation and PSAP secretion in oligodendrocytes contribute to IL-6 secretion, neuroinflammation, and dopaminergic neuron degeneration in an MPTP-induced mouse model [25]. Furthermore, reports of reduced oligodendrocyte densities in the SN and frontal cortex in PD patients, coupled with a decrease in oligodendrocyte precursor cells in the PD brain [26], collectively suggest impaired myelin maintenance and repair mechanisms in individuals with PD [27]. STAT5B plays a key role in regulating the activity and survival of dopaminergic neurons [28]. *STAT5B* knockout mice exhibit significantly reduced brain dopamine levels, increased dopaminergic neuron mortality, and abnormal behaviors, including decreased locomotor activity and anxiety. The overexpression of *STAT5B* enhances the association between dopaminergic neurons and presynaptic neurons, thereby increasing dopaminergic neuron activity and dopamine release [15]. In PD mouse models, activated STAT5B has been shown to inhibit mitochondrial fission and attenuate degenerative damage in dopaminergic neurons [29]. Our results demonstrated that *STAT5B* overexpression significantly improved myelination damage, protected dopaminergic neurons, and ameliorated motor impairments in the MPTP-induced mouse model. The consistent findings across both the in vivo and in vitro experiments further validate the crucial role of STAT5B in mitigating myelin injury and pathological progression in oligodendrocytes. While the MPTP model effectively recapitulates key dopaminergic neurodegeneration and motor dysfunction aspects that are central to PD pathogenesis, it is important to acknowledge its potential for direct neurotoxic effects on various cell types, including oligodendrocytes. Nevertheless, the increasing recognition of myelin dysfunction and oligodendrocyte alterations in human PD brain tissue and other non-MPTP PD models (e.g., alpha-synucleinopathy models) [30,31] suggests that the observed STAT5B-mediated myelin impairment in our study may represent a relevant pathological feature in a broader spectrum of PD etiologies.

MBP is a critical structural component of the central nervous system myelin sheath [32], predominantly expressed in mature oligodendrocytes and essential for myelin formation and maintenance [33,34]. Previous studies have demonstrated that apotransferrin can regulate *MBP* expression in oligodendrocytes through various signaling pathways and TFs [35]. Notably, Sox10 is instrumental in myelination, interacting with Sp1 to promote *MBP* gene transcription [36]. Additionally, p27Kip1 has been shown to enhance *MBP* expression by stabilizing the Sp1 protein and facilitating its binding to the *MBP* promoter [37]. Our study extends these findings by employing a luciferase reporter gene assay to identify STAT5B as a key transcriptional regulator of *MBP*. Furthermore, we demonstrate that *STAT5B* directly binds to the *MBP* promoter, thereby activating its expression and profoundly influencing myelin formation and maintenance.

To investigate the upstream mechanisms contributing to reduced *STAT5B* transcription in oligodendrocytes, we utilized ActD as a transcriptional inhibitor. ActD reduces RNA synthesis by inhibiting RNA polymerase, enabling us to assess *STAT5B* mRNA stability independently of newly synthesized transcripts. Our results indicate that the MPP^+^-induced decrease in *STAT5B* mRNA levels is not primarily due to accelerated mRNA degradation but rather strongly suggests a reduced transcriptional rate. DNA methylation, a key epigenetic mechanism in regulating transcriptional activity, typically suppresses gene expression by methylating promoter regions, thereby hindering transcription factor binding or recruiting repressive complexes [38]. Bioinformatics analysis predicted dense CpG islands within the *STAT5B* promoter, while concurrent qRT-PCR revealed significantly upregulated *DNMT3A* expression following MPP^+^ treatment. This strongly suggests that DNMT3A-mediated DNA methylation contributes to *STAT5B* transcriptional repression, a notion corroborated by studies implicating DNMT3A in PD pathogenesis, such as its role in modulating SNCA expression through targeted methylation [39,40]. Furthermore, the principle of DNMT3A epigenetically silencing crucial glial genes and impairing myelination is not without precedent; for instance, in multiple sclerosis, the hypermethylation of myelination-related genes, including *MBP*, by DNMT3A-implicated mechanisms contributes to oligodendrocyte dysfunction [41]. Our study, therefore, identifies for the first time the DNMT3A-*STAT5B*-*MBP* axis in oligodendrocytes as a direct regulatory pathway, providing compelling evidence for DNMT3A-driven glial epigenetic dysregulation and subsequent myelin injury in PD pathogenesis.

In summary, our comprehensive findings elucidate novel molecular mechanisms underlying oligodendrocyte alterations and myelin damage in the SN of the MPTP-induced mouse model, significantly contributing to the understanding of PD pathogenesis and providing specific gene expression signatures. Our future studies will focus on validating these findings in human PD patient samples and patient-derived cell models. A deeper understanding of oligodendrocyte epigenetic dysregulation during PD progression holds immense promise in revealing novel therapeutic targets, thereby establishing a foundation for the development neuroprotective strategies specifically focused on myelin repair in the treatment of PD.

## Data Availability

The datasets used and/or analyzed during the current study are available from the corresponding author on reasonable request.

## Supplementary Materials

The following supporting information can be downloaded at: https://www.mdpi.com/article/10.3390/cells14151145/s1. Figure S1: The MPTP-induced subacute model mice were successfully constructed; Figure S2: Successful overexpression and knockdown of *STAT5B* in MPP^+^-induced MO3.13 cells; Figure S3: AAV-MAG-OE-STAT5B can effectively overexpress *STAT5B* specifically in oligodendrocytes in the SN of mice; Table S1: The primers and siRNA sequences. Supplementary compressed folder: Western blot analysis with ECL detection showing unprocessed original blots.
