# Supplementary material for: Oligodendrocyte-Specific STAT5B Overexpression Ameliorates Myelin Impairment in Experimental Models of Parkinson’s Disease
Source: Cells. 2025 Jul 25;14(15):1145. doi: 10.3390/cells14151145 (PMC12346846; doi:10.3390/cells14151145)
Supplement: Supplementary file 1 [file cells-14-01145-s001.zip › cells-3733877-supplementary.pdf]

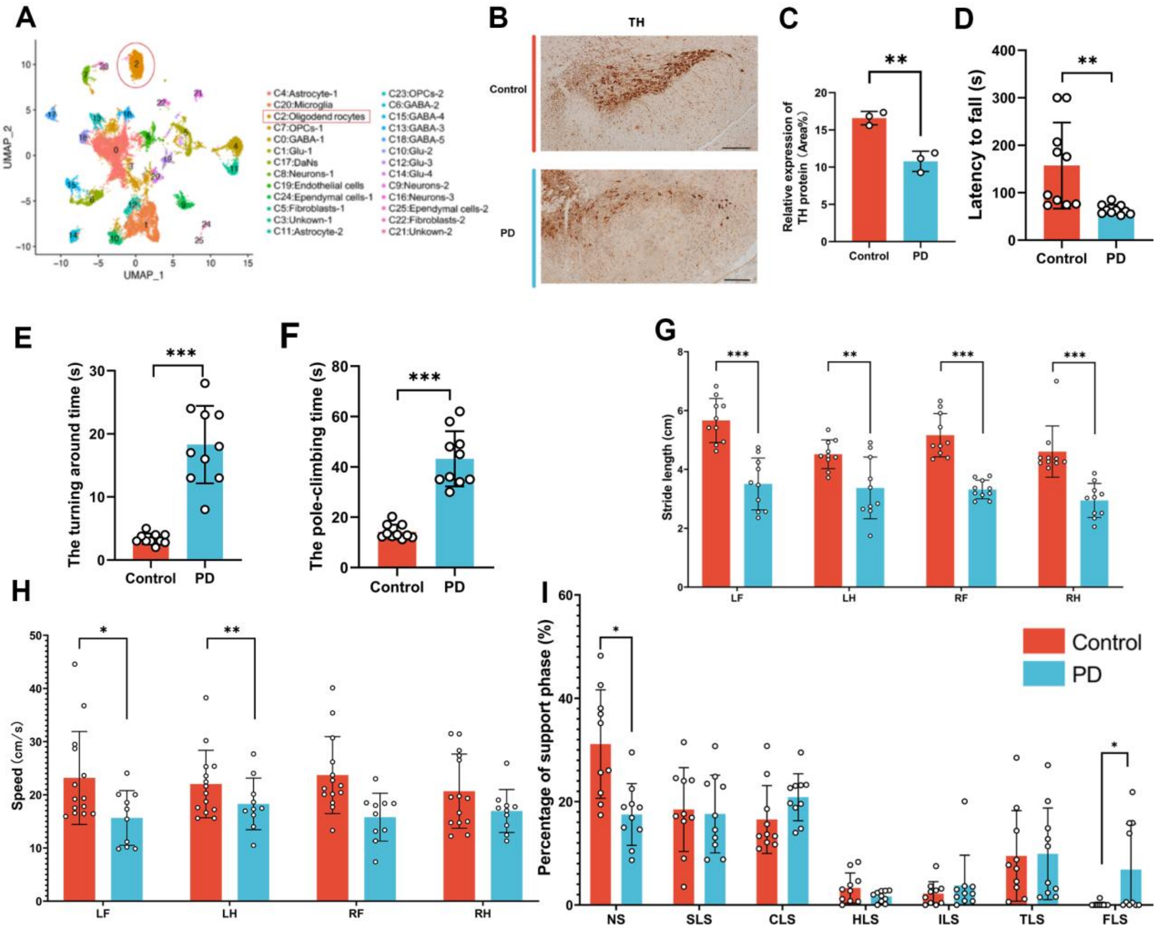


**Supplementary Figure S1. The MPTP-induced subacute model mice were successfully constructed.** (A) UMAP embedding of the 22,983 mouse SN nuclei; colored by cluster. Highlighted to represent the oligodendrocytes cluster. (B) Representative images and (C) quantification of TH$^+$ neurons in the SNpc for each group. Scale bar = 200 μm. (D) Rotarod test results showing fall latency. (E-F) Pole test results showing total time and turn time. (G-I) Gait analysis results showing movement speed, stride length and support time. NS: No Support, SLS: Single-Leg Support, CLS: Contralateral Limb Support, HLS: Homologous Limb Support, ILS: lpsilateral Limb Support, TLS:Three-Limb Support, FLS: Four-Limb Support. The results were expressed as the mean ± SD (n = 10; *, $p < 0.05$; **, $p < 0.01$; ***, $p < 0.001$)

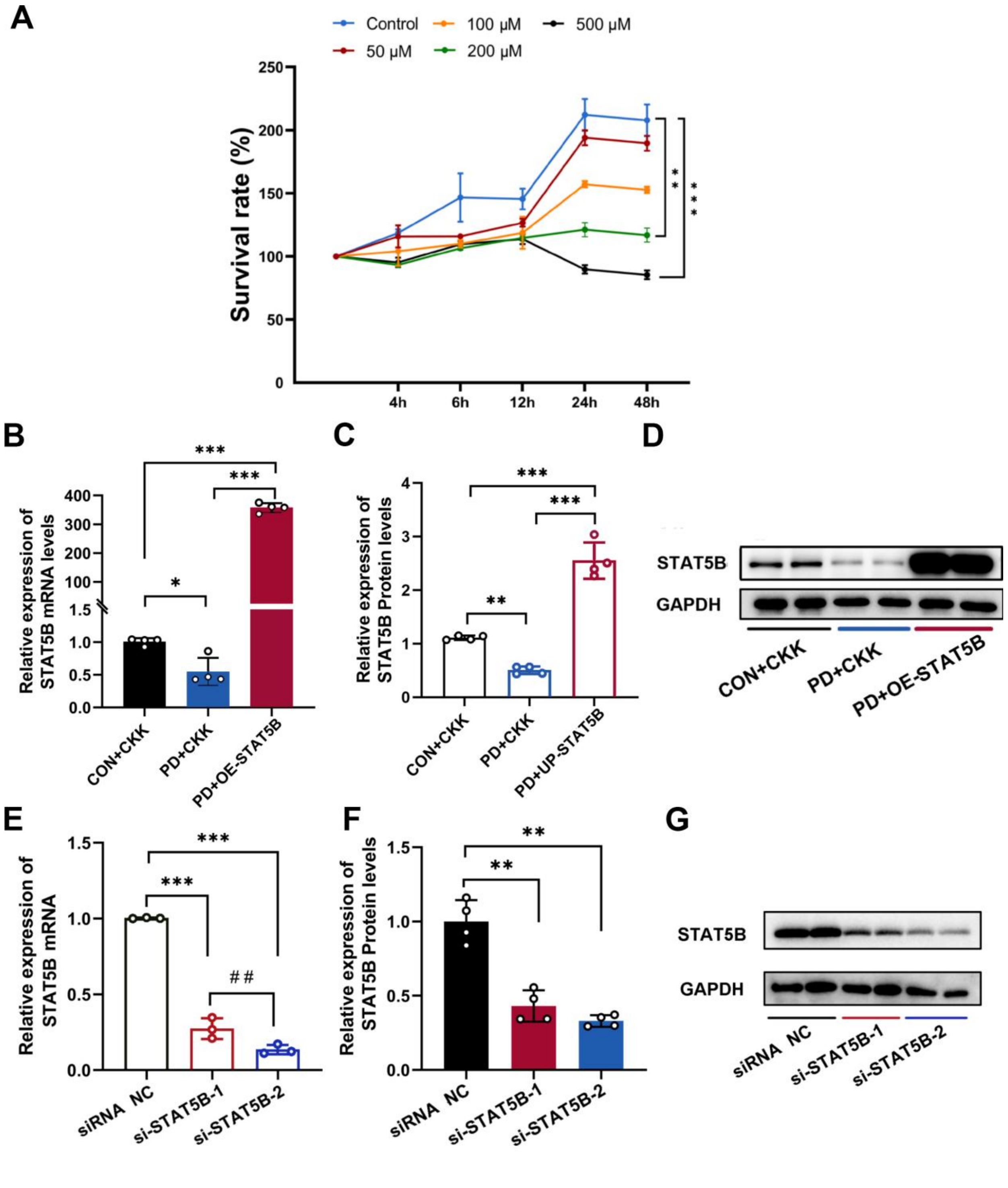


**Supplementary Figure S2. Successful overexpression and Knockdown of STAT5B in MPP⁺-induced MO3.13 cells.** (A) Viability of MO3.13 cells treated with varying concentrations of $MPP^+$ for 4, 6, 12, 24, and 48 h. (B-D) qRT-PCR and western blot analysis of the STAT5B expression in STAT5B-overexpression MO3.13 cells. (E-G) qRT-PCR and western blot analysis of the STAT5B expression in STAT5B-Knockdown MO3.13 cells (*, $p < 0.05$; **, $p < 0.01$; ***, $p < 0.001$).

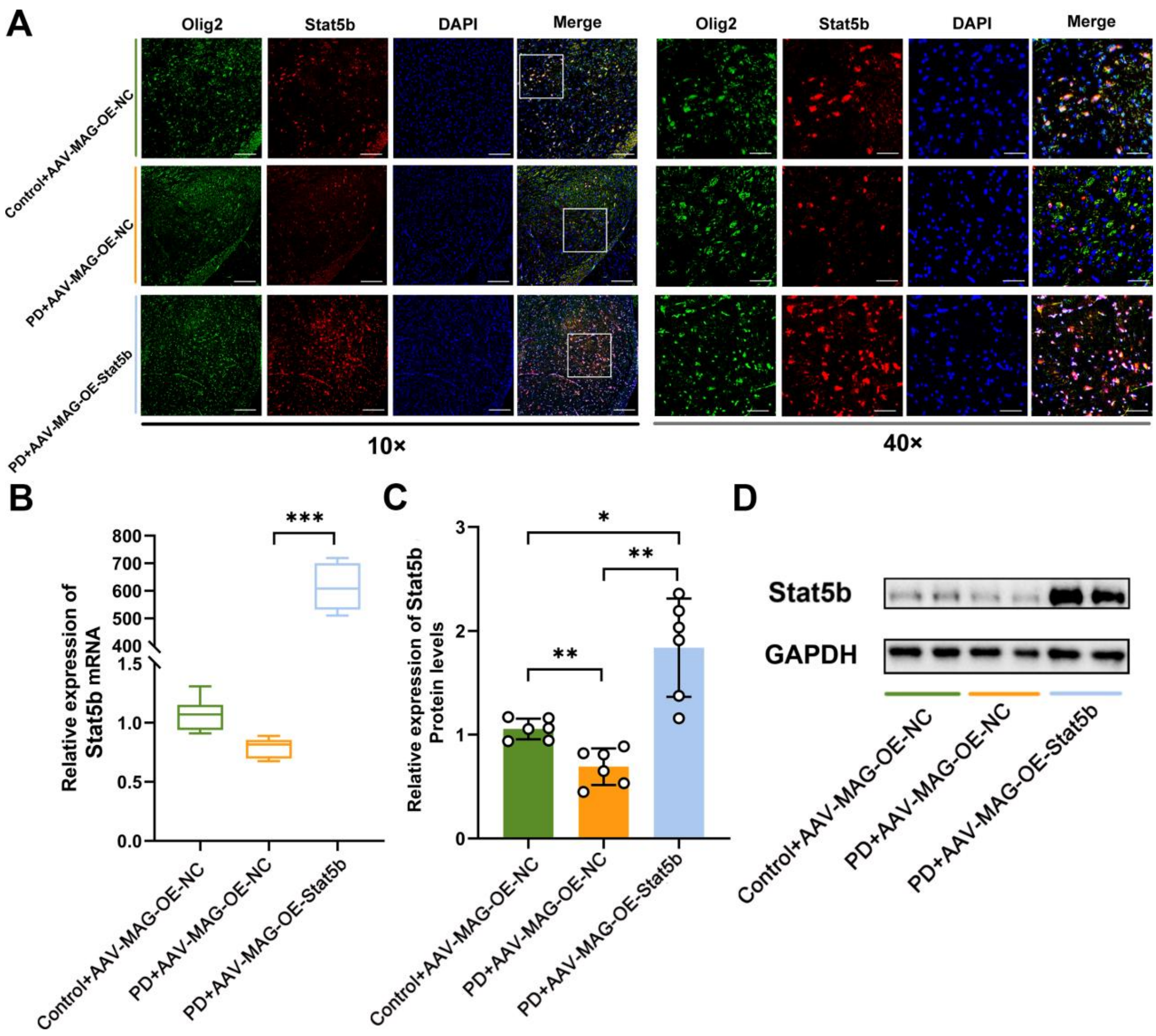


**Supplementary Figure S3. The AAV-MAG-OE-STAT5B can effectively overexpress STAT5B specifically in oligodendrocytes of the SN of mice.** (A) Representative images of brain-stereotaxic AAV virus in the SN for each group (10× Scale bars=200 μm). (B-D) qRT-PCR analysis and western blot analysis of the STAT5B expression in SN of mice. (n = 6; *, $p < 0.05$; **, $p < 0.01$; ***, $p < 0.001$).

Table S1. The primers and siRNA sequences.

| Name | | Primer |
|---|---|---|
| **Hum-STAT5B** | Forward: | 5' AGTTTGATTCTCAGGAAAGAATGT 3' |
| | Reverse: | 5' TCCATCAACAGCTTTAGCAGT 3' |
| **Hum-MBP** | Forward: | 5' CCCTGAGCAGATTTAGCTGG 3' |
| | Reverse: | 5' GAATCCCTTGTGAGCCGATT 3' |
| **Hum-GAPDH** | Forward: | 5' GCAAATTCCATGGCACCGT 3' |
| | Reverse: | 5' TCGCCCCACTTGATTTTGG 3' |
| **Mus-STAT5B** | Forward: | 5' TCAACATCAGCAGCAACCACCTC 3' |
| | Reverse: | 5' TCCATCACGCCATCAAACCACTG 3' |
| **Mus-MBP** | Forward: | 5' GGACCCAAGATGAAAACCCC 3' |
| | Reverse: | 5' CCTTGAATCCCTTGTGAGCC 3' |
| **Hum-DNMT3A** | Forward: | 5' TATTGATGAGCGCACAAGAGAGC 3' |
| | Reverse: | 5' TATTGATGAGCGCACAAGAGAGC 3' |
| **Hum-DNMT1** | Forward: | 5' AGCGAGCGAGCCAGAGATAG 3' |
| | Reverse: | 5' GAGATGCCTGCTTGGTGGAATCC 3' |
| **Hum-DNMT3B** | Forward: | 5' AGCAGCCCTGGAGACTCATTGG 3' |
| | Reverse: | 5' CTGGTTGCGTGTTGTTGGGTTTG 3' |
| **Hum-TET1** | Forward: | 5' GATGACAGAGGTTCTTGCACAT 3' |
| | Reverse: | 5' GATGACAGAGGTTCTTGCACAT 3' |
| **Hum-TET2** | Forward: | 5' GCCTTTGCTCCTGTTGAGTT 3' |
| | Reverse: | 5' ACAAGGCTGCCCTCTAGTTG 3' |
| **Hum-TET3** | Forward: | 5' CACTCCGGAGAAGATCAAGC 3' |
| | Reverse: | 5' GGACAATCCACCCTTCAGAG 3' |
| **STAT5B-2202 Methylation** | Forward: | 5' GTGGGTGTGTGGGGATTTC 3' |
| | Reverse: | 5' CCCTATTTCTTAAAACTTTACTAATACCC 3' |
| **STAT5B-2202 Unmethylation** | Forward: | 5' GTGGGTGTGTGGGGATTTT 3' |
| | Reverse: | 5' CCCTATTTCTTAAAACTTTACTAATACCC 3' |

| | | siRNA |
|---|---|---|
| **Si-STAT5B-1** | Forward: | 5' UCAGAUGCAAGCGUUAUAUTT 3' |
| | Reverse: | 5' AUAUAACGCUUGCAUCUGAUG 3' |
| **Si-STAT5B-2** | Forward: | 5' UGUCCCUGAAACGAAUUAATT 3' |
| | Reverse: | 5' UUAAUUCGUUUCAGGGACAUA 3' |
| **Si-DNMT3A** | Forward: | 5' AGAUGUUCUUUGCCAAUAATT 3' |
| | Reverse: | 5' UUAUUGGCAAAGAACAUCUGG 3' |
